# Fungicide Resistance Management in West Australia’s Wheatbelt

**DOI:** 10.1038/s41597-025-04840-0

**Published:** 2025-03-25

**Authors:** Toto Olita, Billy Sung, Anjana Sharma, Zhanglong Cao, Jackie Mapulanga-Hulston, Mark Gibberd

**Affiliations:** 1 Centre for Crop and Disease Management, Curtin University, Bentley, 6102 Western Australia Australia; 2 Consumer Research Lab, Curtin University, Bentley, 6102 Western Australia Australia; 3 Curtin Law School, Curtin University, Bentley, 6102 Western Australia Australia

**Keywords:** Biotic, Human behaviour

## Abstract

Barley growers from the West Australia Wheatbelt were invited to share information on their fungicide resistance management strategies. The study aimed to identify gaps in growers’ knowledge about issues like fungicide resistance and the objective and/or perceived obstacles and constraints associated with the management of fungal epidemics. To gather this information, we used a case study approach and co-designed the survey in collaboration with industry stakeholders. Socio-economic data was collected using in-depth phone interviews (which made up 82% of the responses) and self-administered questionnaires (which accounted for 18%). The data included both qualitative and quantitative responses. This data covered several aspects: growers’ demographic details, barley production statistics, current knowledge and understanding about fungicide resistance, current agronomic practices, willingness to pay to mitigate fungicide resistance risk, types of fungicide resistance management extension services growers currently use, the reasons for their preferences and additional types of fungicide resistance management extension services growers would like to access in the future.

## Background & Summary

Fungicide resistance is emerging as a significant threat not only to growers but also to the businesses that produce essential plant protection products. The rise of fungicide resistance introduces additional considerations that complicate disease management decision making process^1^. For growers, this involves balancing the rising threat of pesticide resistance against the potential value of their crop and the costs associated with disease management. For fungicide manufacturers, the rise of resistance poses a direct business risk as it diminishes the efficacy of current products in their portfolios. Additionally, developing new pesticide compounds is a lengthy, expensive and complex process. Bringing a new active ingredient to market typically requires over a decade of research and development, along with an investment exceeding USD 300 million^2–4^.

The global regulatory landscape presents additional challenges in managing fungicide resistance^1^. While pesticide regulatory frameworks are essential to protect human health and the environment, without providing suitable alternatives, they can accelerate resistance development by limiting available options and increasing reliance on remaining chemicals. In the European Union, the hazard-based regulatory framework has led to a significant reduction in available fungicides, leading to severe social and economic consequences^4–8^. Similarly, the United States Environmental Protection Agency’s (USEPA) pesticide regulatory framework has been criticised for relying on data provided mainly by pesticide manufacturers during the risk assessment process^9^. This approach raises concerns about potential conflict of interest, as these manufacturers have a vested interest in maintaining market access for their products. Furthermore, this dependency can result in risk assessments that do not reflect real-world implications of pesticide use and resistance. As such, these regulatory frameworks often fail to consider (i) the availability and cost of alternative crop protection measures, (ii) the increased selection pressure on the remaining fungicide mode-of-action groups, and (iii) the practical challenges associated with transitioning to alternative management options. Also, by failing to incorporate independent data and broader stakeholder input, these frameworks leave growers more vulnerable to the impacts of fungicide resistance, further threatening agricultural productivity and sustainability.

The ongoing requirement for the remediation of fungicide resistance highlights the growing tension between pesticide regulatory frameworks and agricultural productivity. Thus, reducing reliance on pesticides means rethinking decades of conventional farming practices. It is therefore important that any techniques utilised in any remediation process combines the necessary cultural, biological and technological methods to control pests with minimal chemical inputs^10,11^. However, the adoption of alternative strategies requires significant investment in research, education and access to innovative tools^12–16^.

Australia is not immune to these problems. Past studies have reported reduced efficacy in major fungicide groups used to manage barley diseases^10,17^, leading to concerns about increased costs of disease management and potential food security threats^18^. This is consistent with the findings which have been reported in the crop protection literature^19–25^. Yet, there is little attention given to the socio-economic consequences associated with fungicide resistance in Australia.

Fungicide resistance also complicates the economic evaluation of crop protection strategies. Growers must assess the potential value of the yield lost to disease and the cost of preventing that loss. To make such an estimate of the trade-off, a grower needs to find that level of inputs at which the gross margin of the enterprise is maximised under limited resources of information, time and capital. Despite these challenges, chemical fungicides are likely to remain popular among growers^1,11,26^. However, with the rise of fungicide resistance, the preference for fungicides creates a dilemma for growers who must balance their desire for chemical pesticides against the long-term sustainability of their crop protection practices.

From initial interaction with the growers as part of the industry initiative known as the “Barley Cohort Project”, it became clear that majority of the growers were deeply concerned about the impact of fungicide resistance on the sustainability of the Australian grains industry. More specifically, concerns centred around understanding the impact on agribusinesses if cheaper fungicides failed to provide adequate protection against fungal pathogens. These interactions highlighted an urgent need to rethink fungicide use, but the challenge associated with measuring the true impact of fungicide resistance remained.

While extensive research exists on the biological aspects of fungicide resistance in Australia’s barley crops, there is a significant gap in understanding the socio-economic dimensions of this challenge. Current data in the literature primarily focuses on the technical aspects such as resistance characterisation and detection, fungicide efficacy and disease management protocols^1,27,28^. However, there is limited information about growers’ fungicide resistance management decision-making process, their understanding of fungicide resistance risks, their sources of fungicide resistance management information and their willingness to invest in preventative measures.

Taken together, the existing literature lacks comprehensive data on how growers access and utilise information about fungicide resistance, their preferred sources of fungicide resistance management advice, and the barriers they face in implementing fungicide resistance management strategies^18^. These knowledge gaps are particularly important in Western Australia’s Wheatbelt, where barley production significantly contributes to the Australian agricultural economy and where fungicide resistance poses an increasing threat to sustainable production^10,17,29^. Therefore, this study sought to identify key factors driving the adoption of fungicide resistance management practices and the resources used by growers to inform their crop protection management decisions.

## Methods

**Fig. 1:**
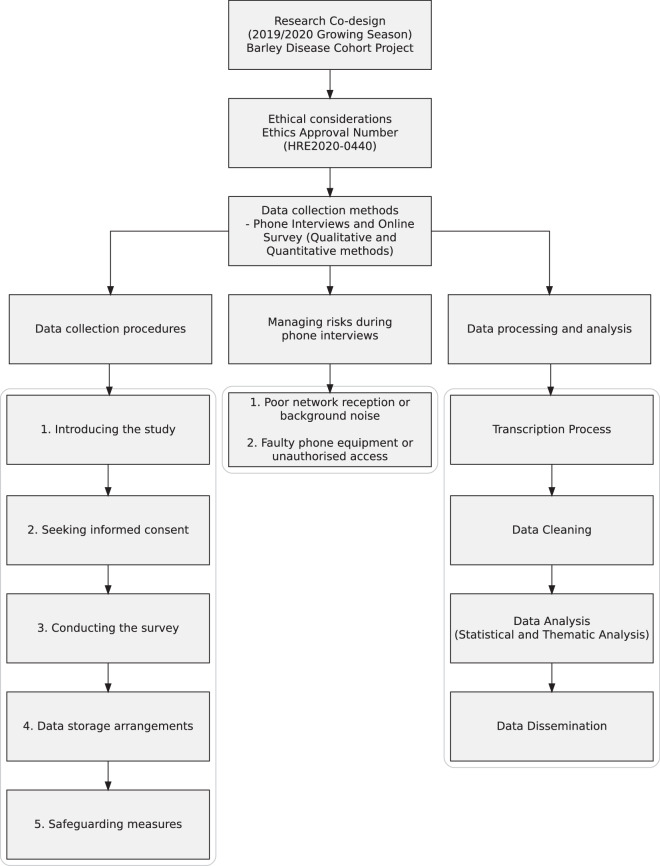
Research activity.

To better understand the challenges associated with fungicide resistance, this study used a case study approach and co-designed the survey in collaboration with grains industry stakeholders. We invited growers from the southwest West Australia’s Wheatbelt region to share their thoughts and insights on what has worked or has not worked for them when managing fungicide resistance issues. The participation occurred through an in-depth phone interview and a self-administered online survey. Figure 1 provides a summary of the research activity.

### Research co-design

The study was conducted during the 2019/2020 growing season as part of the “Barley Disease Cohort Project”. This collaborative research initiative involved growers in southwest West Australia’s Wheatbelt. The study sought to: (i) understand growers’ current knowledge of fungicide resistance, (ii) assess growers’ attitude toward fungicide resistance and its significance to their agribusiness, (iii) evaluate the fungicide resistance management strategies used by growers and (iv) identify the resources used by growers to manage fungicide resistance and the reason for their preferences.

### Ethical considerations

Approval for the study was obtained from Curtin University’s Human Research Ethics Committee (HRE2020-0440) and was performed in accordance with the National Statement on Ethical Conduct in Human Research 2007^30^. When conducting the interviews and online surveys, participants were fully informed about the study’s aims. The participants were to provide informed consent prior to any data collection. Additionally, a participant had the option to withdraw from the study at any point without impacting the relationship with the university.

### Data collection methods

Multiple channels were used to recruit participants to our study. The recruitment materials were disseminated through email lists, industry newsletters, social media platforms, grower group networks and word of mouth. The study accepted interested barley growers and agronomists who voluntarily nominated themselves for participation through the Barley Cohort Project initiative.

We used both qualitative and quantitative data collection methods to enable us to understand how growers are responding to fungicide resistance issues. The qualitative method focused on understanding growers’ personal experiences and what motivates them to change their behaviour in response to fungicide resistance. Here, growers were asked open-ended questions which aimed to elicit detailed insights into the personal experiences and strategies of participants dealing with fungicide resistance issues. On the other hand, the quantitative method involved asking growers to report on their fungicide resistance management practices and perceptions using a Likert scale. Additionally, data on various aspects of their agricultural production were collected to provide context to their fungicide resistance management practices. This included information such as types of barley varieties grown, fungicide application rates and frequencies of fungicide applications, crop used in rotation during 2018/2019 season as well as barley production statistics.

### Data collection procedures

Conducting interviews with growers during the busy planting season requires careful planning and flexibility. Therefore, we made sure to offer flexible timing for our interviews while ensuring the privacy and comfort of participants. Below are the steps we took during the data collection process:

1. *Introducing the study*: The researcher conducting the phone interview first read the information statement to explain the purpose of the study and why the survey was being conducted.
2. *Seeking informed consent*: The researcher then read the information in the consent form and asked the participants to provide consent to participate in the study. Secondly, the researcher also asked the participants whether they consented to have their responses to the survey questions audio recorded. The consent to participate in the study and to have their responses recorded was obtained when the participant indicated “yes”. Participants were informed that their participation was completely voluntary and that they could withdraw their consent at any time of the study.
3. *Conducting the survey*: Participants who agreed to participate in the study were asked the survey questions and their responses were recorded on Qualtrics. Each interview lasted approximately 20–25 minutes.
4. *Data storage arrangements*: For the duration of the project, the digital data (survey, survey results in spreadsheets) were stored on Curtin University’s protected server as per the Data Management Plan in accordance with the Western Australia University Sector Disposal Authority guidelines and Curtin’s Research Data and Primary Materials Policy. The information we collected in this study is to be kept under secure conditions at Curtin University in accordance with Curtin University Information and Communications Technology (ICT) Appropriate Use Policy.
5. *Safeguarding measures*: The server was regularly backed-up by Curtin University. This was to allow the research team to retrieve the data in case of any issues arising during the data collection, data storage and data processing phase of the project.

### Managing risks during phone interviews

Collecting data over the phone during busy planting season presents unique challenges. Therefore, our goal was to gather accurate information while minimising risks that could affect data quality. Below is how we identified potential risks and developed strategies to address them:

#### Challenge 1: Data capture issues due to poor network reception or background noise

##### Potential risk

Considering that our interviews were conducted during the planting season and over the phone, there was a chance that growers may be in the paddocks when contacted. Background noise or poor network reception could cause some responses to be inaudible, leading to incomplete or inaccurate data capture. Despite these risks, phone interviews were still the most effective way to reach more participants in a day, compared to other modes of data collection, such as visiting growers’ sites, which are more time-consuming.

##### Risk mitigation strategies

1. Arranged to have the phone interviews during off-peak times to avoid the possibility of background noises.
2. Asked to reschedule the phone interviews if there were persistent issues with the phone network that may affect the integrity of the data.

#### Challenge 2: Data loss due to faulty phone equipment or unauthorised access

##### Potential risk

Data could be lost if the audio equipment failed to record all the data, is damaged or is accessed without authorisation. This could happen during the interview or while transcribing data to a secured database.

##### Risk mitigation strategies

1. Arranged to have a member of the research team to transcribe the data during the interview as a backup.
2. Implemented a data management plan to mitigate risks associated with unauthorised access. This included but not limited to password protection, locked storage and security clearance to access Curtin University’s data storage drive.
3. Frequently backup all data to Curtin University’s data storage drive to mitigate risks associated with audio-equipment damage.

### Data processing and analysis

Our approach to handling the data focused on accuracy, privacy and extracting valuable insights to better understand growers’ experiences when managing fungicide resistance issues. Below, we highlight the steps in detail:

#### Transcription process

Phone interviews were audio-recorded (with participant consent) and then transcribed. Transcriptions were subsequently uploaded to the Qualtrics platform.

#### Data cleaning

The survey data were cross-checked to ensure consistency with the transcriptions, and any discrepancies with the data directly entered into Qualtrics by the researcher during the interview were corrected. Below, we highlight in more detail:

- Cross-referenced audio recordings with Qualtrics entries.
- Validated postcodes against known Western Australian Wheatbelt regions.
- Cross-verified fungicide application rates against label recommendations.
- Standardised formatting of open-ended responses for consistency (e.g., fungicide product names, variety names).

#### Analysis software

Data analysis was conducted using statistical software (R Program version 4.0.0 or higher)^31^ to analyse quantitative data. For the qualitative data, we used thematic and content analysis to identify common themes related to fungicide resistance management^18^.

#### Data dissemination

To maintain privacy, all collected data was in a non-identified format, meaning no personal information such as participants’ names and addresses were collected. The non-identified data was used to prepare industry reports, professional journal publications and presentations.

## Data Records

### Data and file overview

To maintain both transparency and privacy, our survey data contains anonymous transcriptions of phone interviews and entries from self-administered online questionnaires. The entry for each participant is identified by participant ID and timestamps. The survey response file captures detailed responses to all answered survey questions, providing a rich dataset for understanding growers’ attitudes and behaviour regarding fungicide resistance management. Below, we describe in detail:

*Sample characteristics:*

- Total participants: 137 growers.
- Survey methods: A majority of the participants (82%) completed the survey via phone interviews, while 18% used a self-administered online survey.
- Geographic coverage: Participants were from the southwest region of West Australia’s Wheatbelt.
- Survey Period: Data collection took place between August and December 2020.
*Grower characteristics:*
- Age distribution: The participants’ ages ranged from 22 to 69 years, with an average age of 44 years.
- Industry experience: The participants had between 2 and 54 years of experience in the agriculture industry, with an average of 25 years.
- Farm size: On average, participants cultivated 1122 hectares of barley across 10 paddocks.
*Barley variety diversity:*
- Barley variety distribution: Among the participants, 48% grew two barley varieties, 33% grew one variety, and the remainder grew between three and five varieties.
- The most popular variety was **RGT Planet**, grown by 67% of the participants. Other commonly grown varieties included **Spartacus** (39%), **LaTrobe** (20%), **Rosalind** (18%), **Bass** (13%) and **Flinders** (11%). Less widely adopted varieties (each with adoption rates of 1–6%) were **Banks, Scope, Baudin, Fathom, Litmus, Botler, Granger** and **Hindmarsh**.
*Crop rotation diversity:*
- The most dominant crop in the rotation was **Canola**, grown by 52% of participants. This was followed by **Wheat** (50%), **Barley** (24%) and **Pasture** (23%). Other crops in the rotation, e.g., **Lupins, Oats, Fava Bean** and **Pea**, had lower adoption rates (1–6%).
*Fungicide diversity:*
- The majority of growers (94%) reported using **seed treatment**. Of these, the most popular active group was **fungicide mixtures** (containing multiple modes of action), which was used by 52% of the participants. Additionally, 45% of participants reported using **Group 3** fungicide active seed treatment (DMI – demethylation inhibitors), 11% used **Group 7** fungicide active seed treatment (SDHI – succinate dehydrogenase inhibitors), and only 3% used **Group 11** fungicide active seed treatment (QoI – quinone outside inhibitors).
- When we considered the **first foliar fungicide spray**, 97% of growers reported applying at least one spray. Among them, 80% indicated using **Group 3** fungicide active treatment, 21% used **fungicide active mixtures**, and less than 1% used **Group 11** fungicide active treatment.
- Approximately 53% of the participants reported using a **second foliar fungicide spray**, with 30% using **Group 3** fungicide active treatment and 26% adopting **fungicide mixtures**.
*Fungicide resistance and reduced efficacy observations:*
- Several growers noted possible resistance issues in some of the products that they used. Among those who observed fungicide resistance or reduced efficacy, the majority identified **Group 3** fungicides as affected, particularly **propiconazole** and **tebuconazole**. These findings confirm previous research findings on resistance pressure in Western Australia^17,27^.
- The observed decline in the efficacy of some triazoles highlights the need for a dedicated communication, extension and engagement program focused on fungicide resistance management. This exists as the Australian Fungicide Resistance Extension Network (AFREN), which recommends an integrated disease management approach with an emphasis on varietal selection, non-chemical strategies (such as crop rotation and stubble management), and the judicious use of fungicides through rotating fungicide active and using fungicide mixtures^10^. Furthermore, access to cost-effective and reliable commercial fungicide resistance testing services as well as investments in extension and communication efforts, would benefit the Australian grains industry in managing the emerging threats of resistance^18^.
  *Variable documentation:*Table 1 provides a summary of the variable in the dataset.

**Table 1:** Variables in the dataset.

Variable category	Description	Data type	Response format
Demographics	Age, gender, Education level, Postcode, Farming experience, Grower group membership	Categorical, Numeric	Multiple choice, Numeric values
Farm characteristics	Total barley production area, Soil type, Barley varieties used, Number of barley paddocks, Number of varieties grown, Previous year’s crop	Mixed	Text entry, Numeric values
Management practices	Decision maker for fungicide use, Fungicide usage: Seed treatment product, First foliar treatment product, Second foliar treatment product, Fungicide application timing and rates	Mixed	Multiple choice, Text entry
Economic indicators	Estimated yield potential, Break-even yield estimate, Actual harvest yield, Cost of fungicide treatment, willingness to pay to manage fungicide resistance	Numeric	Continuous values
Disease management	Understanding of fungicide resistance, Perceived causes, control measures, Resistance awareness, Resistance testing done, Reduced efficacy observed, Sources of fungicide resistance knowledge.	Categorical, Text	Text entry, Likert scale (1-7), Multiple choice
*Data access:*
  The data files and survey instrument associated with this work can be accessed through the Research Data Australia’s repository^32^. The dataset includes the following files:
- **1.csv file:** Contains cleaned and anonymised survey responses from both phone interviews and self-administered online questionnaire. No special software is required to open this file.
- **1.xlsx file:** Contains cleaned and anonymised survey responses from both phone interviews and self-administered online questionnaire. Excel software is required to open this file.
- **1.pdf file:** Contains the original survey instrument that was used to conduct the in-depth phone interviews and online survey. The survey questions are classified under the following sections:
  1. Fungicide resistance knowledge assessment.
  2. Disease management on the farm.
  3. Fungicide application pattern on the farm.
  4. Impact of fungicide resistance problem on productivity.
  5. Importance of fungicide resistance management.
  6. Barley production statistics.
  7. Fungicide resistance management practices.
  8. Cost of managing fungicide resistance.
  9. Resources for fungicide resistance management.
  10. Demographic/general questions.
- **1.txt file:** Contains metadata for the dataset, including the principal and co-investigator’s contact details, data collection period, keywords, a short data description, reuse information such as citation details and licence type [*CC BY*].

## Technical Validation

The region of study was southwest of West Australia’s (WA) Wheatbelt with growing seasonal rainfall which falls between April and October^33^. Early interactions with growers through the “Barley Disease Cohort Project” revealed that fungicide resistance was more widespread than expected. The participants involved in the initiative were particularly concerned about fungicide resistance being a long-term threat to a sustainable industry and a worry to their business if cheaper fungicides failed to provide adequate protection against fungal pathogens.

**Fig. 2:**
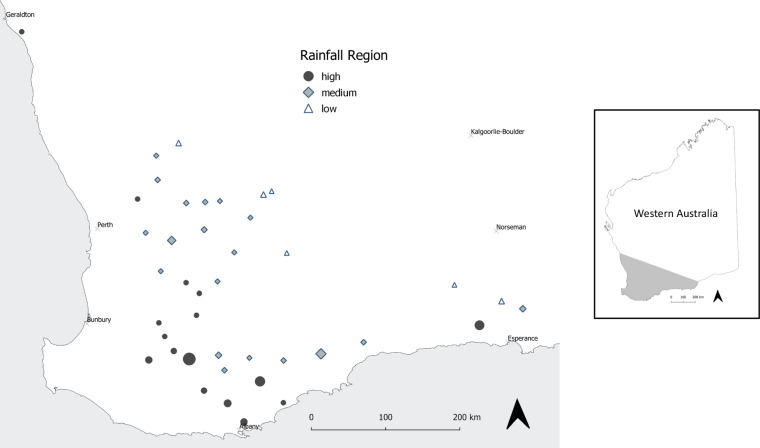
Map distribution of the survey participants categorised according to postcodes and rainfall zones: Low, Medium and High. The size of the symbols denotes the number of participants in each location across the West Australia’s Wheatbelt. Source: Authors.

In order to ensure that the voices of those most affected by fungicide resistance were captured, it was important to gather data that truly reflected the experiences and perspectives of growers. To achieve this, the survey instrument was co-designed with input from industry stakeholders to ensure relevance. This collaborative approach allowed us to include a balanced mix of questions: quantitative and Likert-scale questions for quantifiable insights into growers’ management practices and perceptions, as well as open-ended questions to capture growers’ personal experiences and perceptions about fungicide resistance issues. Figure 2 provides the distribution of survey participants categorised according to postcodes and rainfall zones.

To ensure quality and reliability of the dataset, several key validation steps were undertaken. Below, we describe in detail:

1. Survey instrument validation and Qualtrics implementation

The survey instrument used for data collection was co-designed with industry stakeholders and validated through pilot testing. This collaborative approach ensured that questions were clear, relevant and captured the essential aspects of fungicide resistance management practices. To effectively manage data collection, we used Qualtrics for online questionnaire design, distribution and data collection. The survey instrument was configured in Qualtrics with the following features:

- Optimised for both mobile and web interface.
- Progress indicators using textual cues.
- Validation rules for numerical responses.
- Branch logic for conditional questions.
- Secure data storage ensured participants’ information was protected at all times.
- End-of-survey customised messages.

2. Data collection protocol and quality control

To maintain the integrity of the data during data collection, we implemented several quality control measures:

- Standardised data collection procedures across all the phone interviews and self-administered questionnaires. This included reading the survey questions verbatim during phone interviews to minimise variability.
- Data was collected in a non-identified format to maintain confidentiality.
- Real-time validation during phone interviews with immediate clarification of unclear responses.

3. Data quality assessment

The dataset underwent systematic validation through:

- Mobile compatibility testing prior to releasing the survey instrument.
- Regular data backup protocols in line with the data management plan.
- Cross-verification of transcriptions against original survey responses.
- Standardisation of text entries for consistency (e.g., fungicide product names, variety names).
- Removing typographical errors resulting from data entry.
- Validation of postcodes against known Western Australian Wheatbelt regions.
- Verification of fungicide names and fungicide application rates against a list of fungicides and label recommendations.

4. Analysis framework

The study incorporated both qualitative and quantitative analyses. Below, we discuss in detail:

### Qualitative analysis

The dataset underwent rigorous thematic and content analysis validation through a systematic coding approach. The process involved multiple readings of participant responses to identify emerging themes and patterns in the data. Below are the stages used for thematic and inductive content analysis^18^:

- Initial coding to identify preliminary themes.
- Theme grouping under broader categories.
- Cross-verification of themes across responses.
- Generate insights.

To ensure data reliability, the analysis incorporated:

- Standardised coding procedures across all responses.
- Multiple review cycles to verify theme consistency.
- Cross-referencing of coded themes between researchers.

### Quantitative analysis

The quantitative analysis involved the use of descriptive statistics, multiple factor analysis and multiple linear regression. Below we discuss in detail:

- *Descriptive statistics*
  The analysis began with descriptive statistics, which were used to provide an overview of the dataset and to review quantitative variables for any inconsistencies or anomalies. This included evaluating measures such as mean, median, standard deviation and quartiles as well as distribution patterns across key variables. The descriptive approach enabled an initial understanding of the dataset before proceeding to more advanced analytical methods.
- *Multiple factor analysis (MFA)*
  MFA was implemented to reduce the complexity of the dataset by identifying patterns and relationships between variables. MFA is better suited for datasets with multiple variable groups^34–37^. In our study, the survey questions were normalised by assigning equal weights to related items within each group^29^. This approach ensured that no single group of questions disproportionately influenced the results. The MFA revealed how different variables contributed to variations in outcomes, thereby providing valuable insights into growers’ attitudes and behaviours regarding fungicide resistance management.
- *Multiple linear regression*

After understanding the relationships through MFA, the study employed multiple linear regression to determine which variables influenced key outcomes, such as the return on investment^29^. This analysis allowed us to explore the factors driving growers’ decisions to invest in fungicide resistance management and the economic considerations that affect growers’ willingness to adopt various management practices.

### Limitations and potential biases

We acknowledge several limitations in our dataset:

- Data limitations include:

1. Temporal limitation to 2019/2020 growing season.
2. Limited verification of self-reported yield data since the data collection was anonymous.
3. Limited assessment of fungicide resistance prevalence on farm.
4. Weather and seasonal variations affecting the responses.

- Potential biases include:

1. Self-selection bias from voluntary participation.
2. Recall bias in retrospective questions about past management practices.
3. Geographic representation bias due to phone coverage.

## Usage Notes

Users of this dataset should refer to the corresponding survey response file for detailed insights into the participants’ agricultural production program during the 2019/2020 growing season. This file contains comprehensive data on the barley varieties grown, seed treatment types, foliar fungicide types, fungicide application rates, the growth stages at which fungicides were applied, crops/pasture used in rotation during 2018/2019 season, disease management practices and sources of fungicide resistance management information. For accurate interpretation, we recommend cross-referencing yield data with regional averages and validating any extreme values or outliers before including them in the analysis.

## Data Availability

The data files and survey instrument can be accessed through Research Data Australia’s repository^32^. No custom code was used for the curation or validation of this dataset.
